# Housing prices in China follow Zipf’s law

**DOI:** 10.1371/journal.pone.0324239

**Published:** 2025-05-30

**Authors:** Yalin He, Bailin Zheng, Yue Kai

**Affiliations:** 1 Center of Intelligent Computing and Applied Statistics, School of Mathematics Physics and Statistics, Shanghai University of Engineering Science, Shanghai, China; 2 School of Aerospace Engineering and Applied Mechanics, Tongji University, Shanghai, China; Eötvös Loránd University, HUNGARY

## Abstract

This study verifies that housing prices in China follow Zipf’s law by analyzing the average housing prices in 200 Chinese cities between 2015 and 2023. The Kolmogorov-Smirnov (KS) test further supports this conclusion. Furthermore, the study explores housing price trends in different city tiers and uses rank clocks to reveal price fluctuations, which is not captured by the Zipf’s law. Using nine-year housing price time series data, the ARIMA and ConvLSTM models generate short-term forecasts. The forecasting results are evaluated using various indicators, and KS tests on the predicted prices show a better conformity to Zipf’s law. This paper also builds a dynamic housing price model, offering a new perspective to understand and predict housing price trends. The results of this paper can shed light on the real estate market.

## Introduction

George Zipf first systematically proposed Zipf’s law in 1949, which aimed to describe the distribution of the frequency of word usage in the natural language [1, 2]. Subsequently, Zipf’s law has been widely used in many fields [3–8]. For example, Auerbach found that city sizes follow Zipf’s law [9]. Huberman observed that the distribution of web links adheres to Zipf’s law [10], and Robert Axtell revealed that firm sizes in economics align with Zipf’s law [11]. The Zipf’s law is described by the following formula

$$P(x)\propto x^{-\alpha },$$ (1)

where *x* represents the ranking and α is a positive number which is the exponent of the distribution.

Housing prices are influenced by multiple factors, such as population and income, many of which have been shown to follow Zipf’s law [12–14]. It is natural to explore whether estate prices also conform to this law. There has been some progress in related research. Kaizoji [15] first analyzed the land market in Japan and found that its price distribution conformed to Zipf’s law. Coad [16] studied housing prices in London and confirmed that they conformed to Zipf’s law to a certain extent. Ohnishi *et al*. [17] examined the price distribution of the Japanese real estate market during the bubble period and found that it had a power-law tail. Furthermore, their study demonstrated that US states that experienced real estate bubbles exhibited heavier tail characteristics in their housing price distributions. Blackwell [18] investigated the real estate prices in Charleston County, South Carolina, and also found that they aligned with Zipf’s law to some extent. These studies suggest that prices in some real estate markets may follow Zipf’s law. Therefore, this paper further explores the price distribution characteristics of China’s real estate market.

The distribution of housing prices can also be effectively assessed by comparing the distributions of predicted and actual housing prices. Many scholars have dedicated themselves to researching housing prices, developing a variety of analytical tools and forecasting models [19–21]. These tools leverage historical data to analyze key factors that influence price fluctuations and their interrelationships. For example, the ARCH models by Engle effectively capture housing price dynamics [22]. The Leo Breiman Random Forest model elucidates the complex interplay behind price determinants, achieving precise forecasts [23]. Recently, advances in neural networks and deep learning have further enhanced analytical and predictive capabilities [24–26]. These evolving technologies not only deepen our understanding of the real estate market, but also provide valuable insights for market participants’ decision-making.

As mentioned earlier, significant progress has been made in housing price distribution research, but several key areas remain underexplored. China has the largest housing market worldwide, which has seen remarkable growth since 2015 [27]. Furthermore, while there are abundant researches on Zipf’s law, applying the Zipf’s law to housing price analysis are few, focus on only a few countries and cities. In this paper, we will investigate whether China’s housing prices and their rankings are consistent with Zipf’s law, and also verify the correctness of the conclusions using the KS test.

The purpose of this paper is to explore the distribution of housing prices in the top 200 cities using housing price data from China. Through double logarithmic figures, we preliminarily conclude that housing prices in Chinese cities follow Zipf’s law. To statistically verify this conclusion, this paper uses the KS test for validation. Furthermore, we compare different prediction models and select an appropriate model for short-term housing price forecasting. After analyzing the distribution of the prediction results, we find that it also follows Zipf’s law. Finally, we propose a housing price dynamics model, which aims to better explain the underlying mechanism of housing price distribution and provide theoretical support for housing price research.

## Zipf’s law and KS test

The data for this paper are sourced from the Anjuke website, which has been collecting housing prices and rankings for more than 200 Chinese cities since 2015, accumulating nine years of data to date. All data are available on FigShare (DOI: 10.6084/m9.figshare.26968507.v1). The data were preprocessed to ensure quality. Outliers with excessively high prices have not been removed, considering the specificity of housing price data. This paper uses housing price and ranking data from 2015 to 2023, where housing price data represent the average annual price in each city. To ensure comparability across years, the top 200 cities by housing price ranking are selected for analysis each year.

From the data collected, it can be observed that annual housing prices in Chinese cities have exhibited some volatility. Although the overall trend has shown a steady increase, there has been a decline in recent years. For example, in the first-tier city of Guangzhou, housing prices and rankings from 2015 to 2023 are as follows: RMB 19,855 (ranked 5th), RMB 22,742 (ranked 7th), RMB 28,349 (ranked 7th), RMB 31,831 (ranked 4th), RMB 31,423 (ranked 5th), RMB 30,726 (ranked 5th), RMB 34,690 (ranked 5th), RMB 33,294 (ranked 6th) and RMB 30,953 (ranked 5th). In the case of the fourth-tier city of Hegang, the housing prices and rankings over the same period are as follows. RMB 4,033 (ranked 177th), RMB 3,716 (ranked 236th), RMB 3,922 (ranked 264th), RMB 3,400 (ranked 296th), RMB 3,100 (ranked 349th), RMB 2,022 (ranked 350th), RMB 2,162 (ranked 356th), RMB 2,077 (ranked 357th) and RMB 1,927 (ranked 364th).

In addition, we find that housing prices in most Chinese cities are concentrated below RMB 10,000, with only a few cities showing extremely high prices. Due to the large number of small and medium cities, their housing prices tend to be more centralized, with smaller differences. In contrast, cities at the top of the housing price rankings exhibit significant differences in housing prices. For example, the difference in housing prices between Beijing and Guangzhou exceeds RMB 25,000 in 2023. The gap between the top-ranked and the bottom-ranked city is even more astonishing, at more than RMB 50,000.

**Fig 1:**
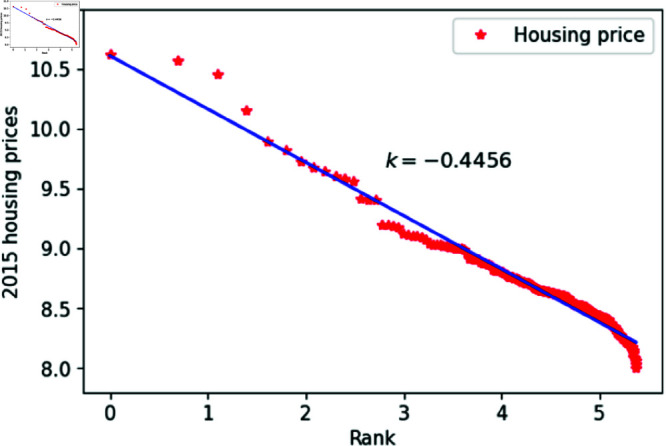
2015 Chinese housing prices and rankings in log–log scale.

**Fig 2:**
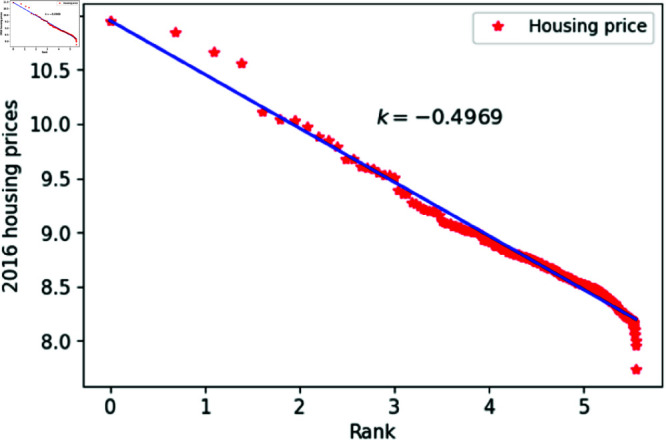
2016 Chinese housing prices and rankings in log–log scale.

**Fig 3:**
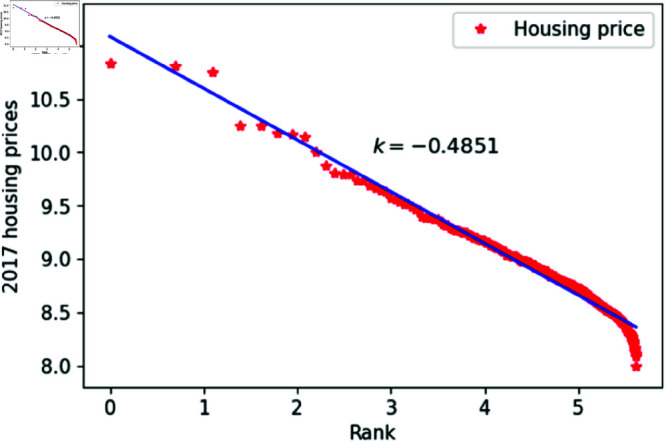
2017 Chinese housing prices and rankings in log–log scale.

**Fig 4:**
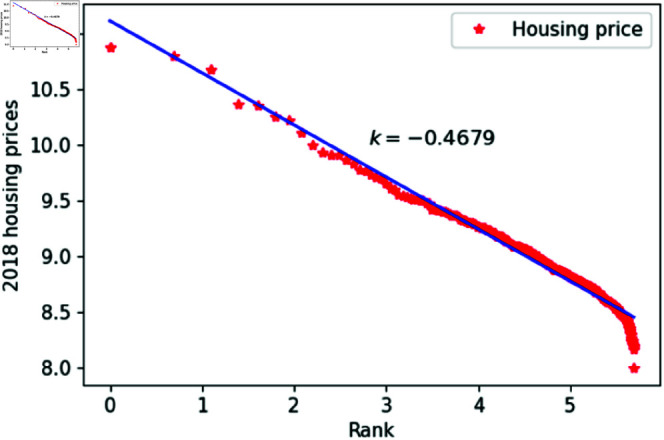
2018 Chinese housing prices and rankings in log–log scale.

**Fig 5:**
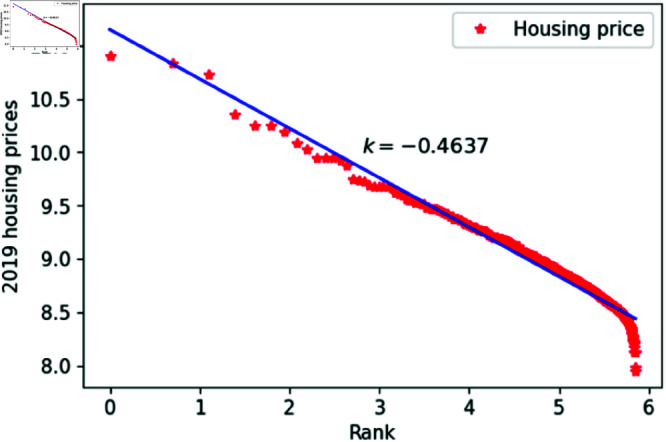
2019 Chinese housing prices and rankings in log–log scale.

**Fig 6:**
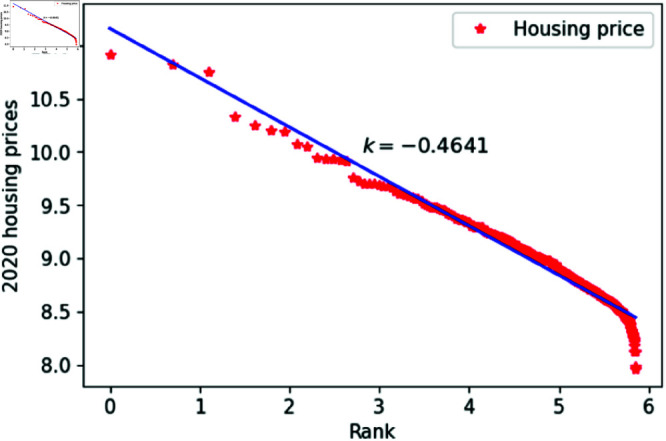
2020 Chinese housing prices and rankings in log–log scale.

**Fig 7:**
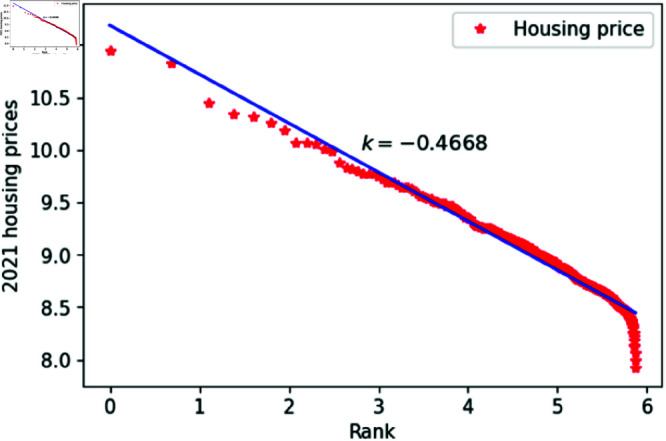
2021 Chinese housing prices and rankings in log–log scale.

**Fig 8:**
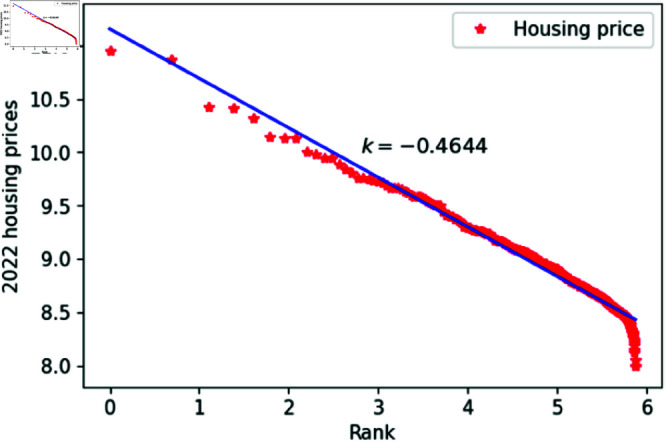
2022 Chinese housing prices and rankings in log–log scale.

**Fig 9:**
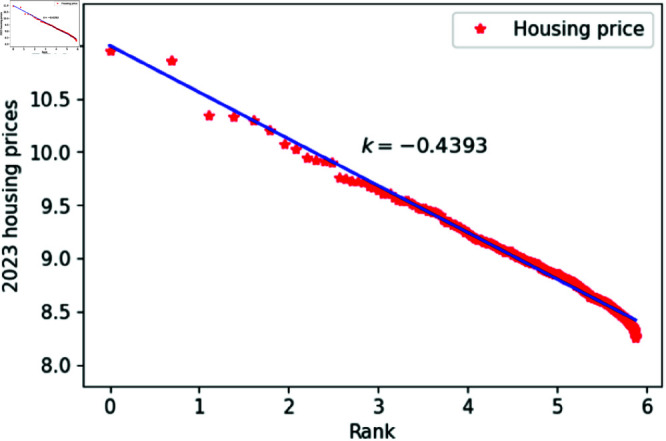
2023 Chinese housing prices and rankings in log–log scale.

Figs 1–9 present a double logarithmic plot of China’s housing prices and rankings for the period 2015 to 2023. The results suggest that housing prices in China largely conform to Zipf’s law [28]. The corresponding exponent values are shown in Table 1. The exponent values in Table 1 indicate that the differences in housing prices among Chinese cities gradually narrowed between 2015 and 2023. This trend reflects the rapid development of China’s real estate market over the past nine years, during which many cities have experienced significant increases in housing prices, in some cases increasing several times. As prices in different cities converge, the gap in overall average city prices naturally decreases. This phenomenon aligns with real-world observations and suggests that China’s real estate market is transitioning from regional differentiation to more balanced development. The R-squared values for Figs 1–9, each exceeding 0.95, suggest that the model closely matches the observed data, indicating excellent goodness of fit.

**Table 1:** Distributional estimates of housing prices in China.

Year	Exponent value	R-squared	p-value	KS statistics
2015	0.4456	0.9979	0.9953	0.02759
2016	0.4969	0.9982	0.9996	0.017466
2017	0.4851	0.9802	0.9997	0.01826
2018	0.4679	0.9768	0.9987	0.02146
2019	0.4637	0.9607	0.9998	0.01459
2020	0.4641	0.9616	0.9998	0.01411
2021	0.4668	0.9654	0.9997	0.01729
2022	0.4644	0.9683	0.9997	0.01776
2023	0.4393	0.9757	0.9998	0.01644

The KS test is performed by comparing the maximum difference between the cumulative distribution function (CDF) of the actual data and the theoretically fitted CDF [29–31]. Specifically, it assesses the maximum deviation between these two CDFs. The KS statistic and the p-value are calculated to determine whether the distribution of Chinese housing prices significantly follows Zipf’s law. The KS test statistic is computed using the following formula

$$K=\max_{x\in \mathbb{R}}|H(x)-D(x)|,$$ (2)

where *H*(*x*) represents the empirical distribution function, *D*(*x*) represents the hypothetical distribution function, and ℝ refers to the dataset we analyze.

The results obtained from the KS test on China’s housing price data from 2015 to 2023 are presented in Table 1. The KS statistic values are all below 0.03, and the p-values are all above 0.9, providing insufficient evidence to reject the hypothesis that China’s housing prices and rankings follow Zipf’s law [32]. Based on these findings, it is reasonable to assume that Zipf’s law accurately describes the data.

## Rank clock

**Fig 10:**
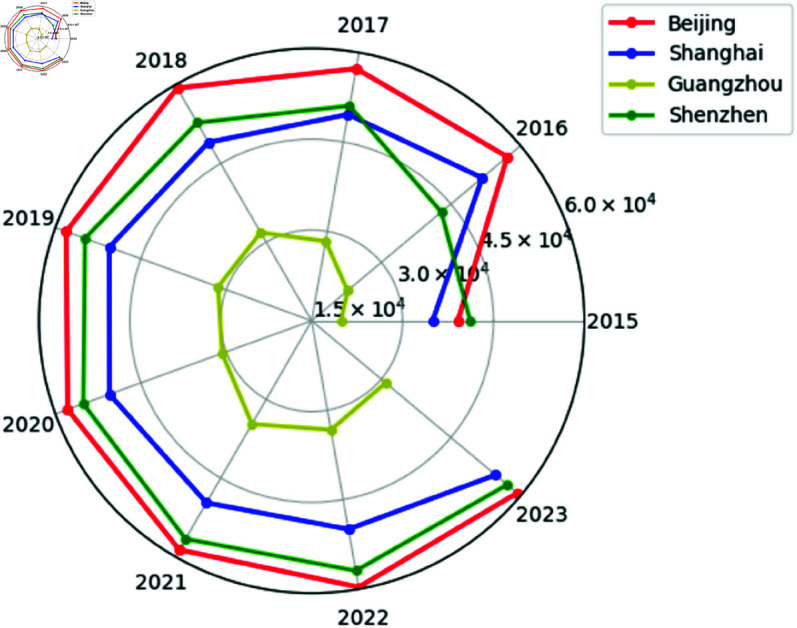
China’s first-tier cities housing prices rank clock.

**Fig 11:**
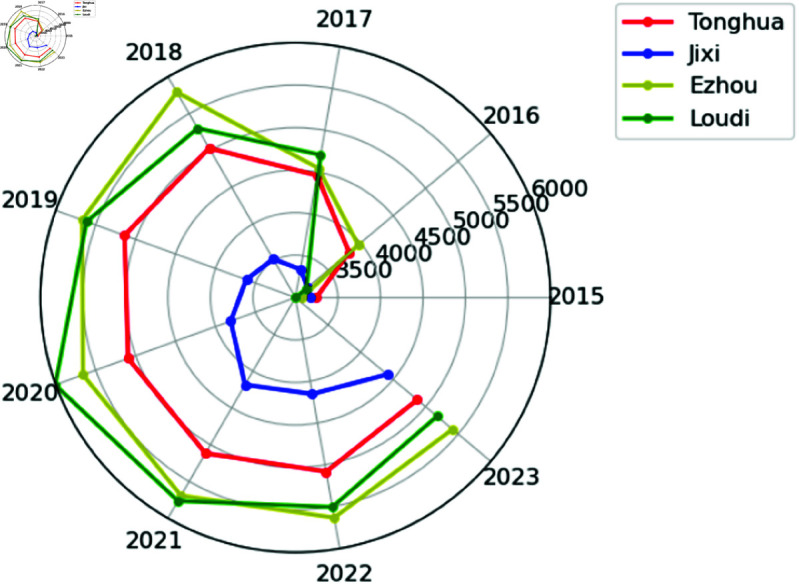
China’s fifth-tier cities housing prices rank clock.

Rank clock offer a method for visualizing dynamic data, especially useful for tracking and displaying changes over time [33]. The rank clock complements Figs 10–11, which might not effectively capture subtle dynamic changes. However, the rank clock excels at capturing and clearly displaying these nuances [34]. For example, the rank clock can plot and analyze the trend of housing prices in Chinese cities over time, showing how prices evolve anticlockwise in polar coordinates. By observing the fluctuations and crossovers of the trajectories, we can visually discern the rise, fall, and stability of housing prices in each city. We selected samples from first-tier cities in China (Beijing, Shanghai, Guangzhou, Shenzhen) and representatives of fifth-tier cities (Tonghua, Jixi, Ezhou, Loudi), displayed in Figs 10–11. Different colored lines are used to distinguish cities, with housing prices represented by the radial distance in polar coordinates.

As shown in Figs 10–11, at the macro level, housing prices in Chinese cities experienced rapid growth from 2015 to 2016. Compared to fifth-tier cities, China’s super first-tier cities not only have more stable housing prices but also demonstrate greater resilience to unexpected events. From a microperspective, housing prices in China’s first-tier cities have entered a relatively stable state after rapid growth and their rankings remain stable. In contrast, housing prices in fifth-tier cities have fluctuated considerably. Comparison of rank clock plots provides a different perspective on housing prices in Chinese cities.

## Short-term forecast of housing prices

In this section, a time series dataset is constructed using data from the top 200 Chinese cities in terms of housing prices between 2015 and 2023, with 70% of the data used for the training set and 30% for the test set. We found that the traditional LSTM model may struggle to capture the underlying trends in the data. To improve the model’s prediction performance, we constructed a ConvLSTM model by incorporating 2D convolution and merging layers into the traditional LSTM model. Furthermore, since our time series data do not exhibit seasonality, we selected the ARIMA model for comparative forecasting [35, 36].

First, we present the results of the ConvLSTM model. After using Keras Tuner and performing several experimental optimizations [37], we finally selected the following parameter configurations: the number of LSTM layers is 3, with 256 kernels in the first layer, 128 kernels in the second layer and 64 kernels in the third layer. All kernels have a size of (1, 1), and the merge window size is (1, 1, 1, 1). The specific configurations are as follows: the number of filters in each ConvLSTM2D layer ranges from 32 to 256, in increments of 32, as determined by Keras Tuner optimization. The final fully connected Dense layer has 1 neuron. The dropout rate for each layer ranges from 0.2 to 0.5, with the exact values determined by Keras Tuner. After testing the model in the test set, the ${\text{R}}^{2}$ and precision were 0.9851 and 97.64%, respectively. For the ARIMA model, we debugged and arrived at the ARIMA (2, 1, 2) configuration. After testing the ARIMA model on the test set, the ${\text{R}}^{2}$ and accuracy were 0.9943 and 96.84%, respectively.

**Fig 12:**
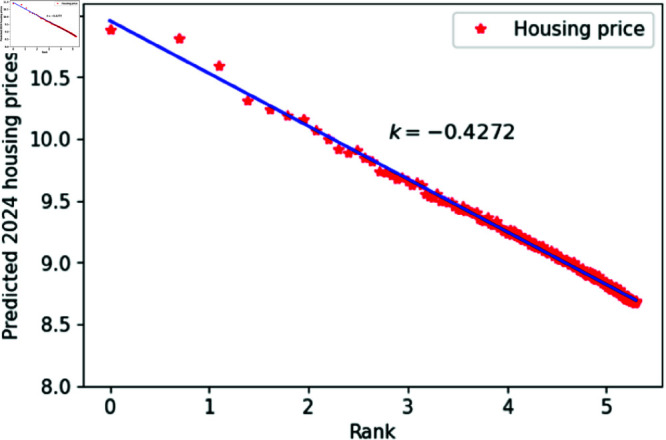
2024 Predicted Chinese housing prices and rankings in log–log scale.

**Fig 13:**
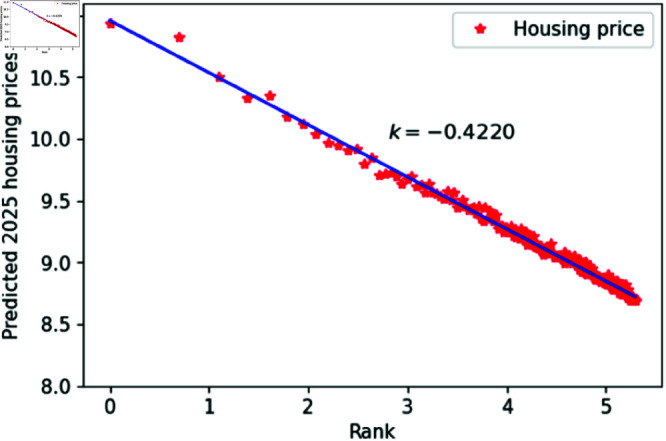
2025 Predicted Chinese housing prices and rankings in log–log scale.

**Fig 14:**
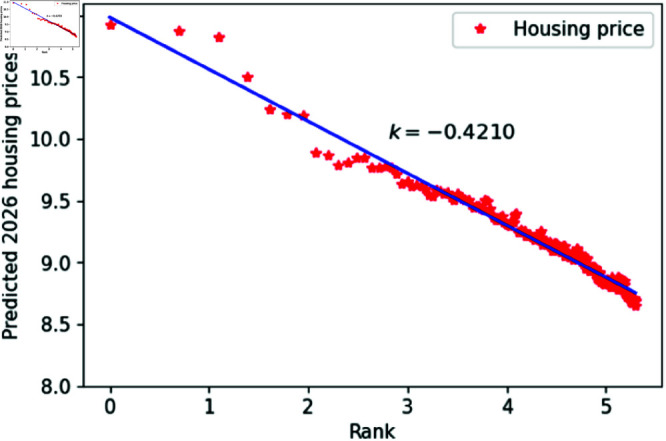
2026 Predicted Chinese housing prices and rankings in log–log scale.

From the overall results, the ConvLSTM model outperforms the ARIMA model in many aspects of predicting housing prices in Chinese cities. Therefore, we selected the ConvLSTM model for future predictions of housing prices. Consequently, we used the model to predict housing prices in 200 Chinese cities for the next three years, plotting the double logarithmic figure and performing the KS test. The results shown in Figs 12–14. and Table 2, indicate that the predicted housing prices for the next three years conform well to Zipf’s law, further supporting the hypothesis that housing prices and their rankings in Chinese cities follow Zipf’s law.

**Table 2:** Distributional estimation of forecasted housing prices in China.

Year	Exponent value	R-squared	p-value	KS statistics
2024	0.4272	0.9963	0.9987	0.01637
2025	0.4220	0.9924	0.9679	0.01608
2026	0.4210	0.9769	0.8967	0.01625

## Housing price dynamic model

In economics, it is a common assumption that logarithmic changes in prices satisfy certain laws [38], we assume that the log change in housing prices in Chinese cities associated with housing price rankings satisfies the following conditions

logP(i)−logP(i+1)=u(i), (3)

where *i* represents the ranking of housing prices in China (the city with the highest housing price *i* = 1, the city with the second highest housing price *i* = 2, and so on), *u*(*i*) is consistently positive and monotonically decreasing, so we can write it as $\frac{1}{h(i)}$, where *h*(*i*) is consistently positive and monotonically increasing

$$\log P(i)-\log P(i+1)=\frac{1}{h(i)},$$ (4)

we assume *i* = *nl*, when *n* is an extremely large value, *l* is an extremely small value, we can written as

$$\log P(nl)-\log P(nl+1)=\frac{1}{h(nl)}.$$ (5)

We discuss the case where *P*(*i*) is a homogeneous function, and we get P(nl)=P(n)P(l), which follows

$$\log[P(n)P(l)]-\log\left[P(n)P\left(l+\frac{1}{n}\right)\right]=\frac{1}{h(nl)},$$ (6)

suppose *h*(*i*) is linear function, let *h*(*i*) = *h*_1_*nl*, then Eq. 6 can be reduced into the following equation

$$\log P(l)-\log P\left(l+\frac{1}{n}\right)=\frac{1}{h_{1}\;nl},$$ (7)

multiply both sides of the equation by *n*, we can derive

$$\frac{\log P(l)-\log P\left(l+\frac{1}{n}\right)}{\frac{1}{n}}=\frac{n}{h_{1}nl}=\frac{1}{h_{1}l},$$ (8)

when *n* tends to infinity, we can get

$$\frac{\partial \log P(l)}{\partial l}=\frac{1}{h_{1}l},$$ (9)

which yields,

$$\log P(l)=\frac{1}{h_{1}}(\log l+\log C),$$ (10)

we can get solution

$$P(l)=Cl^{-\frac{1}{h_{1}}}.$$ (11)

**Fig 15:**
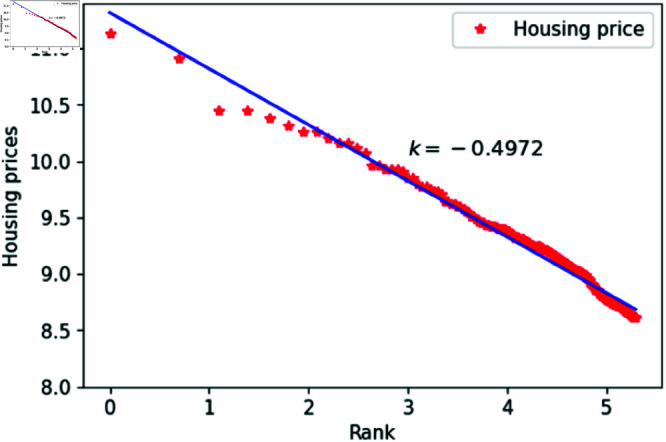
When *h*_1_ = 1.83.

**Fig 16:**
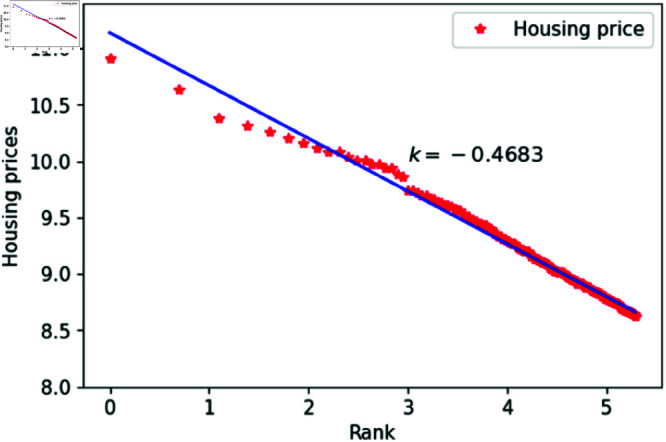
When *h*_1_ = 1.84.

**Fig 17:**
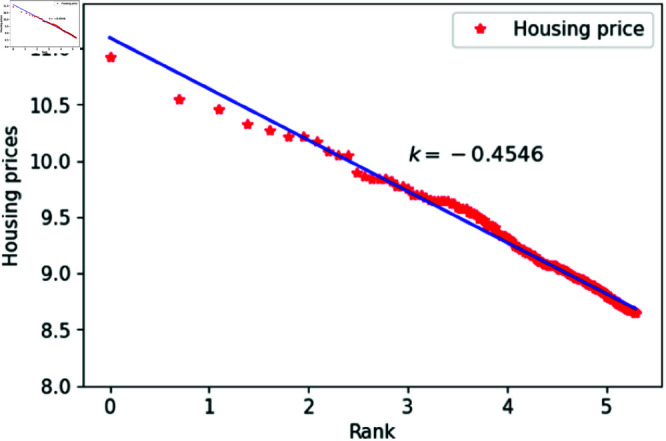
When *h*_1_ = 1.85.

**Fig 18:**
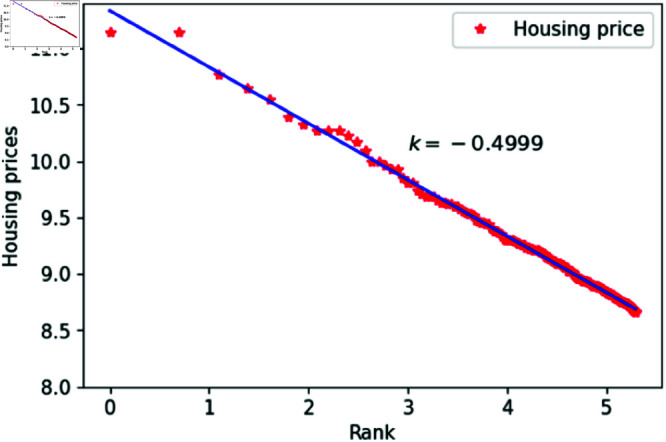
When *h*_1_ = 1.86.

**Fig 19:**
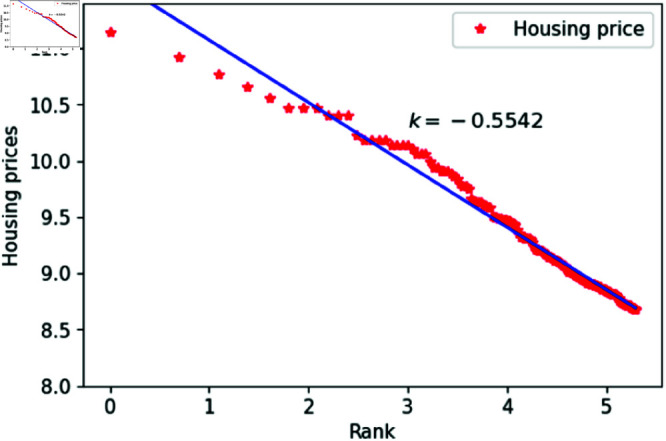
When *h*_1_ = 1.87.

**Fig 20:**
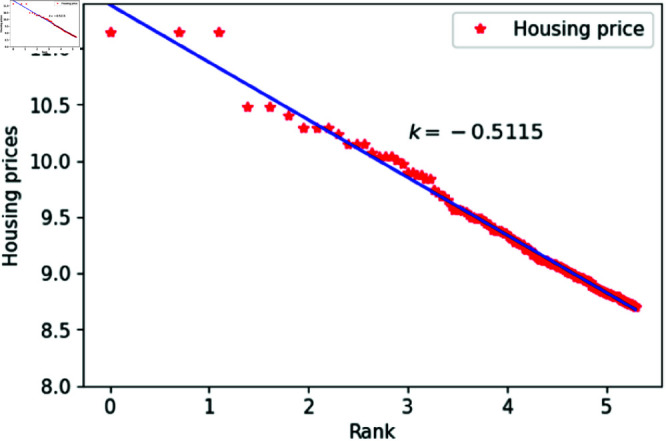
When *h*_1_ = 1.88.

We can think of *l* as a quantity related to ranking, randomly assigned an integer value between 1 and 200. The constant *h*_1_ varies between 1.83 and 1.88, specifically taking values such as 1.83, 1.84, 1.85, 1.86, 1.87 and 1.88. The constant *c* is set to 1,000,00 to calculate housing prices that closely match the actual data. After fitting the data, the results are shown in Figs 15–20, with the corresponding exponent values and R-square values listed in Table 3. The results in Figs 15–20 and Table 3 shown that the housing prices obtained by Eq. 11 follow Zipf’s law.

**Table 3:** Fitting of housing price distributions calculated by the dynamic model.

${\text{h}}_{\text{1}}$	Exponent value	R-squared
1.83	0.4972	0.9640
1.84	0.4683	0.9641
1.85	0.4546	0.9801
1.86	0.4999	0.9941
1.87	0.5542	0.9935
1.88	0.5115	0.9892

## Conclusion

In this paper, we focus on the housing prices and ranking of Chinese cities from 2015 to 2023. By analyzing the annual housing prices and rankings using double logarithmic figures, we initially determined that they follow Zipf’s law. Further, we use the KS test to calculate the KS statistic and p-value, which verify that the housing prices and rankings of Chinese cities indeed follow Zipf’s law. In addition, we employ the rank clock to explore trends and fluctuations in housing prices in China’s first-tier cities and some fifth-tier cities, revealing characteristics of housing prices that are not captured in the previous figure. To further investigate whether the housing prices and rankings of Chinese cities will continue to follow Zipf’s law in the future, we use various models to make short-term forecasts for housing prices. Upon comparing the prediction metrics, we finally select the ConvLSTM model for short-term forecasting. The KS tests of the predicted results confirm that the foretasted housing prices also closely adhere to Zipf’s law. Finally, we construct a housing price dynamic model to provide new perspectives on housing price forecasting and research.
